# Increased Ventral Striatal Volume in College-Aged Binge Drinkers

**DOI:** 10.1371/journal.pone.0074164

**Published:** 2013-09-27

**Authors:** Nicholas A. Howell, Yulia Worbe, Iris Lange, Roger Tait, Michael Irvine, Paula Banca, Neil A. Harrison, Edward T. Bullmore, William D. Hutchison, Valerie Voon

**Affiliations:** 1 Toronto Western Research Institute, Division of Brain Imaging and Behaviour — Systems Neuroscience, Toronto Western Hospital, University Health Network, Toronto, Canada; 2 Institute of Medical Science, University of Toronto, Toronto, Ontario, Canada; 3 Department of Psychiatry, University of Cambridge, Cambridge, United Kingdom; 4 Behavioural and Clinical Neurosciences Institute, University of Cambridge, Cambridge, United Kingdom; 5 Department of Psychology, University Of Cambridge, Cambridge, United Kingdom; 6 Institute for Biomedical Imaging and Life Sciences, University of Coimbra, Portugal; 7 Brighton and Sussex Medical School, University of Sussex, Brighton, United Kingdom; 8 Cambridgeshire and Peterborough NHS Foundation Trust, Cambridge, United Kingdom; 9 GlaxoSmithKline, Clinical Unit Cambridge, Addenbrooke's Hospital, Cambridge, United Kingdom; 10 NIHR Cambridge Biomedical Research Centre, Cambridge, United Kingdom; 11 Departments of Surgery & Physiology, University of Toronto, Toronto, Canada; Bellvitge Biomedical Research Institute-IDIBELL, Spain

## Abstract

**Background:**

Binge drinking is a serious public health issue associated with cognitive, physiological, and anatomical differences from healthy individuals. No studies, however, have reported subcortical grey matter differences in this population. To address this, we compared the grey matter volumes of college-age binge drinkers and healthy controls, focusing on the ventral striatum, hippocampus and amygdala.

**Method:**

T1-weighted images of 19 binge drinkers and 19 healthy volunteers were analyzed using voxel-based morphometry. Structural data were also covaried with Alcohol Use Disorders Identification Test (AUDIT) scores. Cluster-extent threshold and small volume corrections were both used to analyze imaging data.

**Results:**

Binge drinkers had significantly larger ventral striatal grey matter volumes compared to controls. There were no between group differences in hippocampal or amygdalar volume. Ventral striatal, amygdalar, and hippocampal volumes were also negatively related to AUDIT scores across groups.

**Conclusions:**

Our findings stand in contrast to the lower ventral striatal volume previously observed in more severe forms of alcohol use disorders, suggesting that college-age binge drinkers may represent a distinct population from those groups. These findings may instead represent early sequelae, compensatory effects of repeated binge and withdrawal, or an endophenotypic risk factor.

## Introduction

Binge consumption of alcohol is a serious and pervasive public health issue across many countries and demographic groups. Binge drinking is especially common among college-aged individuals, with a prevalence reported between 43–58.5% and 32–54% in men and women, respectively, in the United Kingdom and United States [1]–[3]. It is associated with significant social and personal costs, such as physical injury, motor vehicle accidents, sexually transmitted diseases, unintended pregnancies, and medical complications such as high blood pressure, stroke, and other cardiovascular diseases [4].

Research on anatomical differences in BD is comparatively limited. A study of adolescent BD has found a negative association between the maximum number of drinks during a binge and cerebellar grey and white matter volume [5]. Adolescent BD have also been shown to have decreased white matter coherence compared to healthy volunteers [6]. One study has addressed alterations in young-adult BD grey matter volume, focusing on cortical thickness. Female BD had thicker left frontal cortices whereas male BD had thinner left frontal cortices compared to gender matched controls [7]. Prefrontal cortical decline has been observed in adolescents suffering from alcohol use disorders (AUD) as well [8]. While no studies have reported group differences in subcortical grey matter volume between BD and healthy controls, left hippocampal volume differences were observed between adolescent alcohol users and dual marijuana and alcohol users [9]. It was also reported that smaller left hippocampal volumes were correlated with alcohol abuse/dependence symptoms, and that adolescent alcohol users had a larger right > left hippocampal asymmetry than that of controls and users of both alcohol and marijuana. Chronic, non-pathological alcohol consumption can also cause volumetric changes in adults. Increased alcohol consumption was negatively correlated with total brain volume in a large community-based sample and total lifetime alcohol use was correlated specifically with decreased frontal cortical grey matter volume in a sample of adult Japanese men [10], [11].

Studies of grey matter differences in persons with more severe AUD have identified decreased volumes in subcortical structures. For instance, abstinent persons with AUD had decreased amygdala and nucleus accumbens volumes compared to controls, with accumbens volume also positively correlated with length of abstinence [12]. The observation of decreased grey matter volume in AUD has been replicated in the ventral striatum [13], [14], the amygdala [12], [14], and the hippocampus [14]–[17], including in adolescent AUD [18]. In summary, there is broad agreement that, across the lifespan, alcohol use and misuse can cause volumetric changes in the frontal cortices as well as subcortical structures. However, as neuroanatomical development continues through adolescence into early adulthood, the changes that occur in earlier or later periods of life, or as a result of more severe AUD conditions may not generalize to subclinical populations of different ages. Alcohol-related changes in the volume of subcortical structures have not, to date, been addressed in a young-adult population with milder forms of alcohol misuse. Therefore, in this study, we compared college-aged BD and healthy volunteers using voxel-based morphometry, focusing on subcortical regions implicated in previous reports on AUD and adolescent drinking. We hypothesized that BD would have lower grey matter volume in the ventral striatum, amygdala, and hippocampus relative to healthy volunteers. In contrast to our hypothesis, we show here that ventral striatal volume is greater in BD compared to controls.

## Methods

### Participants

The study was approved by the University of Cambridge Research Ethics Committee. Nineteen BD and 19 healthy volunteers, matched for gender and education, participated in the study (Table 1). Written informed consent was obtained from all participants. Participants were recruited from the Cambridgeshire region by local advertisements in both community- and university-based settings. Healthy volunteers were also recruited from the Behavioural and Clinical Neurosciences Institute healthy volunteer panel. The criteria used for binge drinking were based on the National Institute on Alcoholism and Alcohol Abuse diagnostic criteria [19]: consumption of ≥5 drinks (8 units) and ≥4 drinks (6 units) in a 2-hour period (for males and females, respectively) at least once a week for the last three months. BD also indicated that their drinking was motivated by a desire to get drunk. Participants were included if they were greater than 18 years old, had no history of regular or current use of other substances, and were free from any major psychiatric disorders (assessed using the Mini International Neuropsychiatric Inventory) [20]. Those presenting with major neurological illness, head injury, or who were not suitable for the MRI environment were not included in the study. All participants were asked to refrain from alcohol consumption at least 24 hours before scanning and underwent a urine drug screen and an alcohol breathalyzer test.

**Table 1:** Demographic and behavioral data for healthy volunteers and binge drinkers.

Group	Sex (M/F)	Age	Smoking History	NART	AUDIT	BDI	STAI	UPPS-P
**Healthy Volunteers (n = 19)**	7/12	24.63 (4.40)	0	117.67 (3.87)	3.21 (2.67)‡	7.08 (7.10)†	41.28 (11.56)†	124.83(23.89)†
**Binge Drinkers (n = 19)**	7/12	22.95 (3.41)	2	116.53 (5.19)	15.79 (6.20)	8.11 (6.58)	41.00 (11.50)	139.95 (27.04)
**T/Chi-square (P-value)**		1.32 (0.195)	2.11 (0.486)	0.77 (0.451)	−7.10 (P<0.001)	−0.45 (0.653)	0.07 (0.942)	−1.80 (0.080)

AUDIT: Alcohol Use Disorders Identification Test, BDI: Beck Depression Inventory, NART: National Adult Reading Test, STAI: Spielberger Trait Anxiety Inventory. Standard deviations in brackets except where noted otherwise.

All p-values reported are for 2-tailed independent samples t-tests, equal variances not assumed, except smoking history, which was tested with Fisher's Exact Test.

† : Missing values from one participant.

‡ : Missing values from 5 participants.

### Data acquisition

Participants completed a questionnaire on past and current use of other substances, the Alcohol Use Disorders Identification Test (AUDIT) [21], Beck Depression Inventory [22], IQ as assessed by the National Adult Reading Test (NART) [23], Spielberger Trait Anxiety Inventory (STAI) [24], and UPPS-P impulsivity scale [25]. Participants were scanned using a Siemens 3T Tim Trio scanner (Siemens Medical System Systems, Erlangen, Germany) with a 32 channel head coil at the Wolfson Brain Imaging Center at the University of Cambridge. T1-weighted structural images were acquired using the following sequence (TR  = 2300 ms; TE  = 2.98 ms; matrix: 240×256×176 mm, voxel size 1×1×1 mm).

### Data processing

The 3D T1-weighted images were preprocessed with Statistical Parametric Mapping software (SPM8) (http://www.fil.ion.ucl.ac.uk/spm). The images were reoriented, aligning the origin approximately to the anterior commissure. New Segment was used to segment the images by tissue type (CSF, white matter, and grey matter) using a tissue probability map to assign voxels a probability of belonging to one of these groups. The total intracranial volume for each participant was estimated on the basis of the summed volume of these tissue classes. DARTEL, a diffeomorphic method, was used to generate a sample-specific template for the non-linear deformation of the grey and white matter images [26]. This template was then registered to the tissue probability maps using an affine transformation in the warping procedure. The images were registered in ICBM 152 MNI space. All images were smoothed using a 10 mm FWHM isotropic Gaussian kernel in the final normalization step.

### Statistical analyses

Full factorial general linear models (GLM) were used to compare the parameter estimates of grey matter concentrations from BD and healthy volunteer groups. Models controlling for age and AUDIT score were also tested. All GLMs were corrected for the total intracranial volumes of the participants using proportional scaling and an explicit mask, creating a binarized image from the SPM brainmask template using ImCalc. Whole brain voxel-wise group comparisons were performed using a cluster extent threshold correction. The cluster extent threshold correction was calculated at 19 voxels at P<0.001 whole brain uncorrected, which corrected for multiple comparisons at P<0.05 assuming an individual-voxel Type I error of P = 0.01 [27]. Anatomical localization of the cluster peaks was obtained through the MNI coordinates AAL atlas structures [28].

Given our *a priori* hypothesis that particular regions would be affected in BD, groups were also compared using small volume corrections (SVC) to the GLM, targeting the regions identified in the introduction. To define these regions, the AAL atlas in the WFUPickAtlas SPM Toolbox [28] was used for amygdalar and hippocampal SVC corrections. The ventral striatum regional correction template was hand drawn using MRIcro using the delineations of Martinez and colleagues [29] and has been used in previous studies [30]. A family-wise error (FWE) threshold of P<0.05 within the SVC was used to determine significance of results from these tests. Additional GLMs with AUDIT, UPPS-P (total score), and UPPS-P (sensation seeking subscale) scores as covariates were tested within these SVC regions.

All demographic and behavioral data were compared with two-tailed, independent samples T-tests without assuming equal variances, except smoking history, which was assessed with Fisher's Exact Test. Correlations with AUDIT score were performed by extracting the peak-voxel parameter estimates from SVC regions of interest. Statistical analyses on demographic, behavioural, and correlational data were performed using IBM SPSS Statistics 20 (IBM Corporation, Armonk, NY).

## Results

### Participants

Nineteen BD and 19 healthy volunteers were recruited. There were no significant differences in participant characteristics, depression, and anxiety scores, although there was a trend toward a difference in impulsivity scores (Table 1). As expected, BD subjects had higher AUDIT scores compared to healthy volunteers (*t*_(26.43)_  = −7.90, p<0.001).

### VBM data

**Figure 1:**
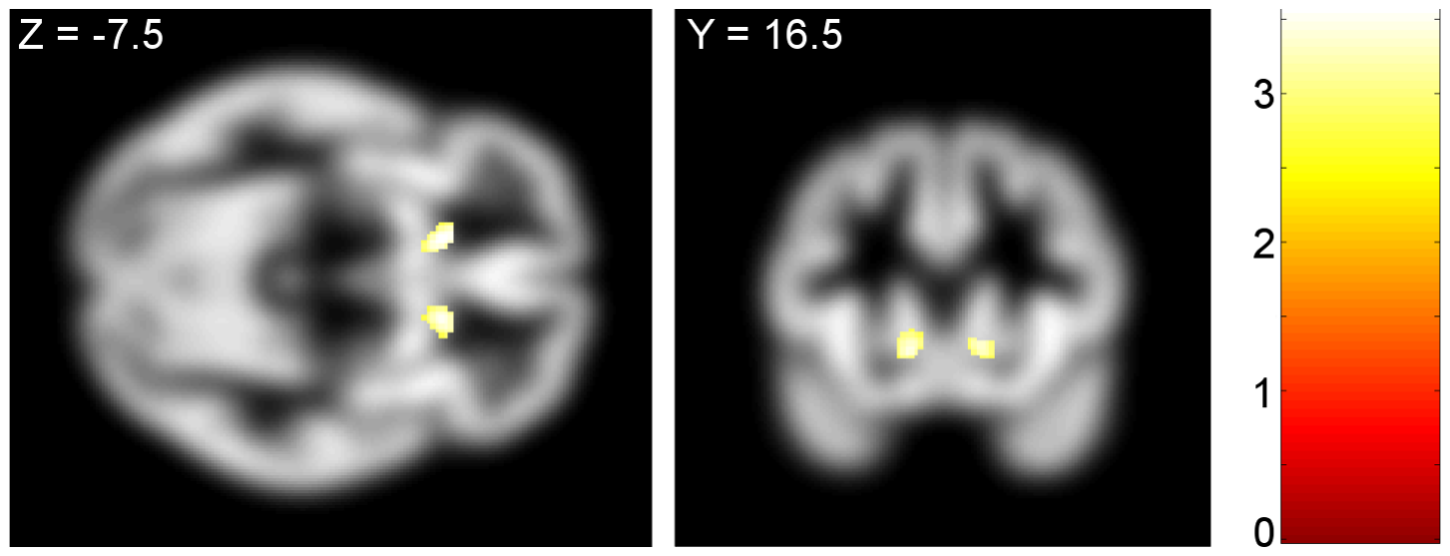
Binge drinkers' ventral striatal grey matter volume is enlarged compared to controls. The SPM image shows greater bilateral ventral striatal grey matter volume in binge drinkers versus controls. Left side: Montreal Neurological Institute peak coordinates (*x, y, z*; mm): −12.0, 19.5, −6.0; Z = 3.57, FWE-corrected P = 0.030; cluster size  = 52. Right side: (*x, y, z*): 13.5, 19.5, −6.0; Z = 3.28, FWE-corrected P = 0.030; cluster size  = 49. The image is shown at whole brain uncorrected P<0.005, cluster size >0, overlaid on the mean group grey matter image, and masked according to the region of interest. Voxel-wise t-values are color coded according to legend. The images are displayed in neurological convention.

Compared to healthy volunteers, BD had greater bilateral ventral striatum volume (whole brain cluster extent threshold corrected) (Table 2). This difference was also apparent using an SVC analysis focusing on the ventral striatum (Figure 1). The cluster threshold analysis also indicated greater grey matter volume in the left thalamus and right lingual gyrus of binge drinkers. Cluster extent threshold analysis showed that healthy volunteers had significantly greater grey matter volume in the right precuneus compared to BD (Table 3). There were no group differences in hippocampal or amygdala volumes under an SVC or whole brain analysis. The observed difference in ventral striatum grey matter volume remained significant when controlling for age in the SVC analysis (Left side: Montreal Neurological Institute (*x, y, z*; mm) peak coordinates: −12.0, 19.5, −6.0; Z = 3.29, FWE-corrected P = 0.029; cluster size  = 48. Right side: 14.0, 20.0, −6.0; Z = 3.17; FWE-corrected P = 0.041; cluster size  = 13). The ventral striatum group differences also remained significant controlling for AUDIT score in the SVC analysis (Left side: −4.0, 14.0, −2.0; Z = 4.23, FWE-corrected P = 0.001; cluster size  = 210. Right side: 12.0, 18.0, −8.0; Z = 4.25; FWE-corrected P = 0.001; cluster size  = 357).

**Table 2:** Regions exceeding cluster extent threshold (binge drinkers > healthy volunteers).

Structure	MNI Coordinates (*x, y, z*)	Z-Score	Cluster Extent	P-Value
**L Ventral striatum**	(−12, 20, −6)	3.28	56	P<0.001
**R Ventral Striatum**	(14, 20, −6)	3.28	55	P<0.001
**L Thalamus**	(−14, −7, 6)	3.38	31	P<0.001
**R Lingual gyrus**	(28, −67, −2)	3.77	146	P<0.001

MNI: Montreal Neurological Institute, L: Left, R: Right.

Results reported in the following tables using cluster extent thresholds were generated from statistical parametric maps at a threshold of k = 19 and P<0.001 whole brain uncorrected. Anatomical localizations were performed using the AAL atlas (see Methods for more details).

**Table 3:** Regions exceeding cluster extent threshold (healthy volunteers > binge drinkers).

Structure	MNI Coordinates (*x, y, z*)	Z-Score	Cluster Extent	P-Value
**R Precuneus**	(16.5, −66, 21)	3.59	367	P<0.001
**n.a.**	(36, −64.5, 10.5)	4.37	84	P<0.001

MNI: Montreal Neurological Institute, L: Left, R: Right.

Results reported in the following tables using cluster extent thresholds were generated from statistical parametric maps at a threshold of k = 19 and P<0.001 whole brain uncorrected. Anatomical localizations were performed using the AAL atlas (see Methods for more details).

**Figure 2:**
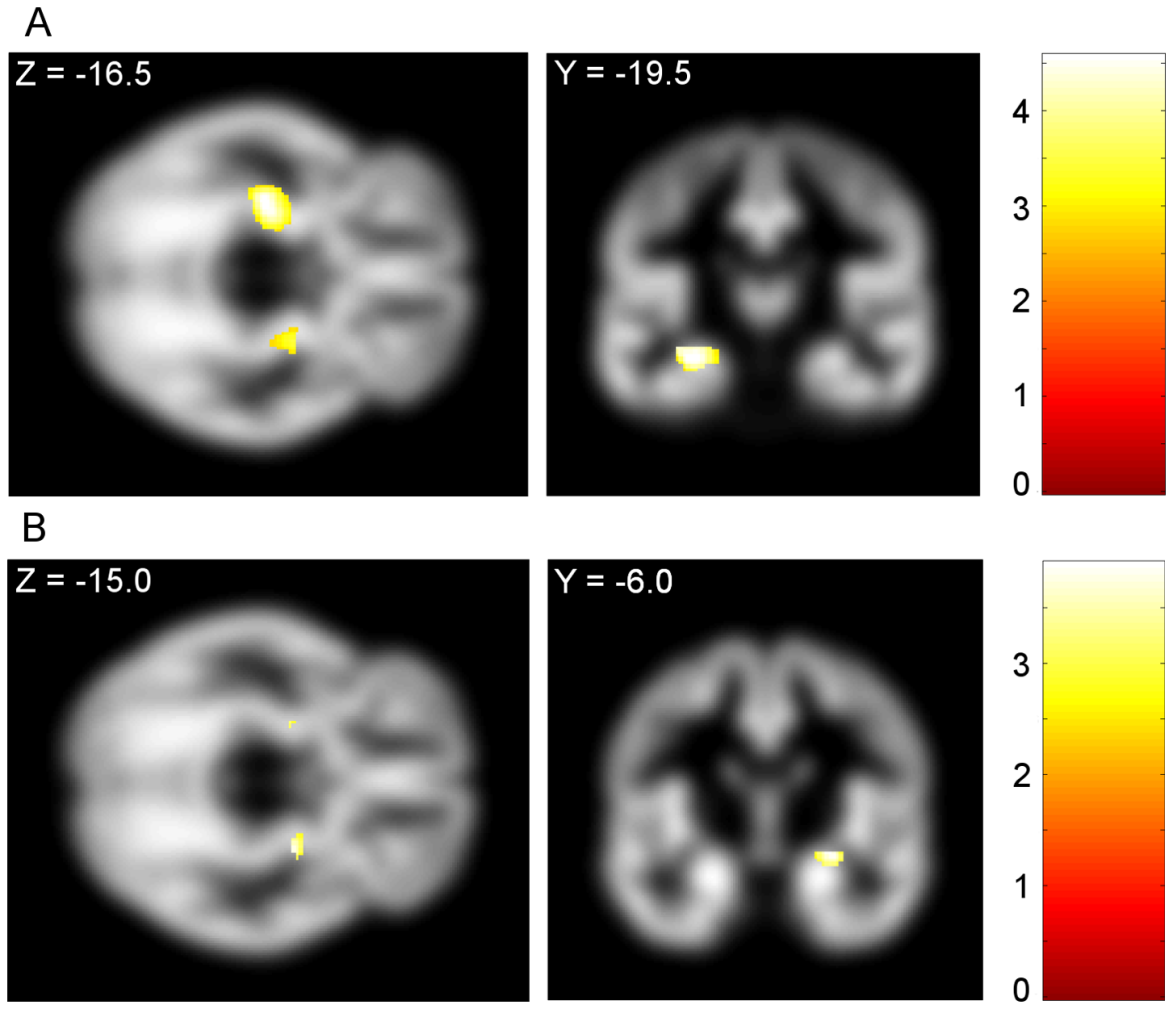
Alcohol Use Disorders Identification Test scores are negatively correlated with hipppcampus and amygdala grey matter volume. A) Regions of hippocampus and B) amygdala negatively correlated with Alcohol Use Disorders Identification Test scores across groups. Hippocampus: Left side: −30.0, −19.5, −16.5; Z = 3.93, FWE-corrected P = 0.009; cluster size  = 289. Amygdala: Left side: −22.5, −9.0, −12.0; Z = 3.08, FWE-corrected  = 0.037; cluster size  = 4. Right side: 27.0, −7.5, −13.5; Z = 3.46, FWE-corrected P = 0.012; cluster size  = 43. All images are shown at whole brain uncorrected P<0.005, cluster size >0, overlaid on the mean group grey matter image, and masked by the atlas delineations of these structures in neurological convention. Voxel-wise t-values are color coded according to legend.

**Figure 3:**
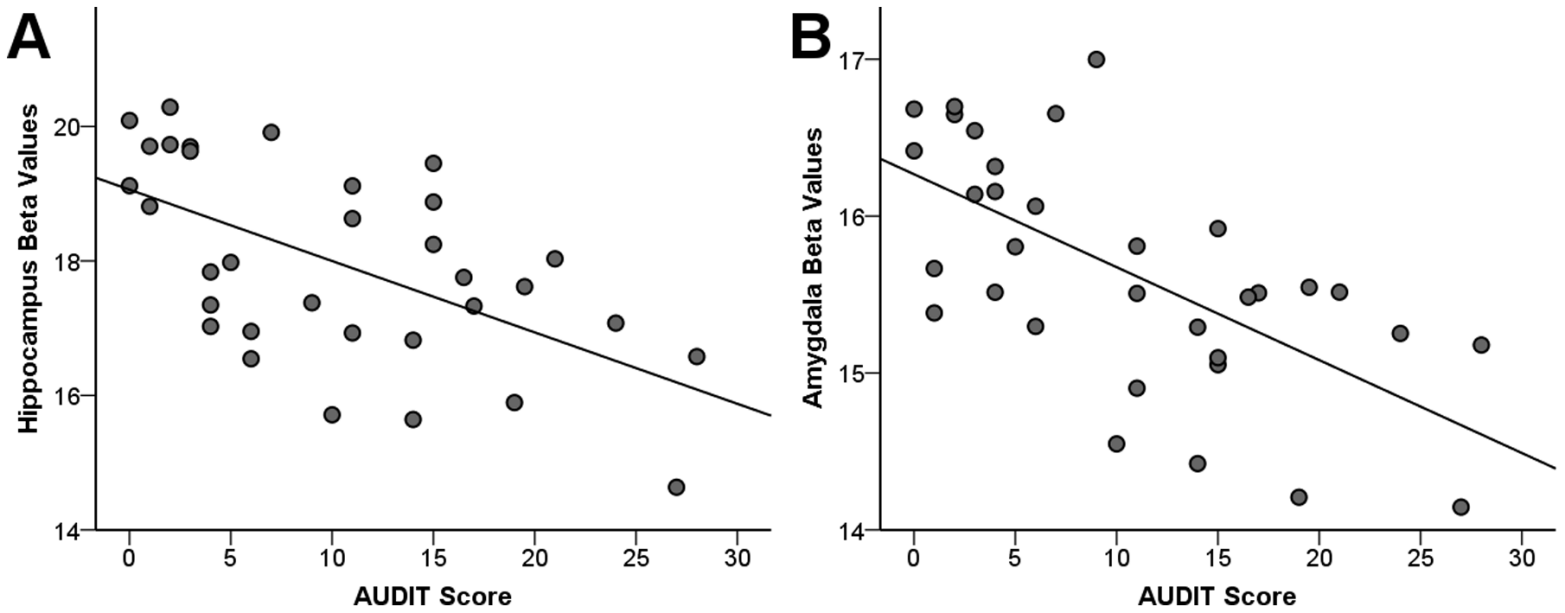
Alcohol Use Disorders Identification Test scores are negatively correlated with hippocampus and amygdala grey matter volume. Plots of peak voxel beta values versus Alcohol Use Disorders Identification Test (AUDIT) scores. Hippocampus and amygdala peak voxel parameter estimates were significantly negatively correlated with AUDIT score (R^2^  = 0.336, P<0.001, and R^2^  = 0.410, P<0.001, respectively).

The AUDIT score was also assessed as a covariate. There were no regions correlating with the AUDIT score at a whole brain FWE P<0.05 threshold. Left ventral striatal grey matter volume was negatively correlated with AUDIT scores on SVC analysis (Left side: −4.5, 13.5, −1.5; Z = 3.51, FWE-corrected P = 0.016; cluster size  = 14. Right side: 12.0, 18.0, −7.5; Z = 3.16, FWE-corrected P = 0.043; cluster size  = 3). Additional negative correlations between AUDIT scores and both left hippocampal and bilateral amygdalar grey matter volume were also observed (Figure 2). The correlations between AUDIT score and both hippocampal and amygdalar peak voxel parameter estimates were significant (Hippocampus: R^2^  = 0.336, P<0.001; Amygdala: R^2^  = 0.410, P = 0.001) (Figure 3). The UPPS-P total score and sensation seeking subscales were also assessed as covariates, but no significant main effects on grey matter volume in target regions were observed.

## Discussion

Anatomical MRI studies in persons with more severe forms of AUD, such as alcohol dependence, commonly demonstrate decreased striatal, amygdalar and hippocampal volumes [12]–[14], an effect which reverses with prolonged abstinence [12], [13]. In contrast to our hypothesis, we show that BD have greater bilateral ventral striatal volume compared to healthy controls. In line with the reports in AUD, we show that across all participants, ventral striatal, hippocampal and amygdalar volumes were inversely correlated to AUDIT scores, an index of alcohol misuse severity. The ventral striatal difference was also present controlling for age and AUDIT score, further supporting an underlying difference between the two groups. Thus, our findings of greater ventral striatal volume in BD participants cannot be explained by the severity of alcohol use. The inverse correlation with AUDIT scores and the known decrease in ventral striatal volume with more severe alcohol misuse argues against our findings being related to a state effect of chronic alcohol consumption. The negative correlations between AUDIT score and amygdalar and hippocampal grey matter volume is also in agreement with results reported from AUD patients. Notably, the left hippocampus was also identified as affected by alcohol in previous studies of adolescent alcohol exposure. Medina and colleagues found that left hippocampal volume and right > left hippocampal asymmetry were each negatively associated with symptoms of alcohol misuse [9]. In the case of the hippocampus, these changes may relate to the inhibition of neurogenesis established to take place in this structure. In rodent models of heavy consumption of alcohol, dentate gyrus neural progenitor cells were inhibited in proliferation and survival [31]. While neurogenesis in the amygdala has attracted less attention, there is evidence supporting its occurrence [32]. Intriguingly, recent work has also suggested that hippocampal neurogenesis may be, in part, regulated by signals from the basolateral amygdala [33]. Moreover, lesions to the basolateral amygdala were found to decrease the activation of new hippocampal neurons after expression of a fear memory. While neuroimaging data is not of sufficient resolution to distinguish the subnuclei of the human amygdala, it is interesting to speculate that the reductions in amygdala and hippocampal grey matter volume observed here and reported previously are mediated by these effects. Indeed, this link may underlie the impaired fear conditioning seen in young binge drinkers and reduced amygdalar and hippocampal plasticity observed in rodent models of binge drinking [34]. In summary, these findings may represent early sequelae, compensatory effects of repeated binge and withdrawal, or alternatively a trait effect. Moreover, the reductions in amygdalar and hippocampal grey matter volume may be caused by alcohol-induced inhibitions of neurogenesis. Prospective longitudinal studies or studies in unaffected family members are indicated to differentiate some of these possibilities.

Of note, our study was conducted in college-aged young adults. Previous experiments have largely focused on adults and adolescents. The present findings, therefore, provide unique data on the bridging period between these two age groups. Our hippocampal results agree with a large body of research studying individuals throughout the life span that have shown hippocampal anomalies associated with alcohol consumption. In contrast, however, the enlargement of ventral striatum grey matter in BD is at odds with previous reports from adult samples. This may relate to the particular developmental stage of our participants. Imaging studies have shown that striatal volumes decline in grey matter between adolescence and young adulthood [35]. This present result may, therefore, represent relative neuroanatomical immaturity in this population. Future work directly comparing adolescent, young-adult, and adult BD would be of great interest in uncovering the interaction between drinking behaviours and development.

The ventral striatum plays a key role in motivational and reward processes. It serves as the major input structure for the limbic loop of cortico-striatal circuitry, receiving afferents from sites such as the orbitofrontal and anterior cingulate cortices, the amygdala and anterior insula [36]. It additionally has reciprocal connections with dopaminergic cells in the ventral tegmental area. In rodent models, alcohol is self-administered into the nucleus accumbens shell, suggesting that it may in part act directly at the ventral striatum [37]. Microdialysis experiments have further shown that dopamine release in the ventral striatum accompanies alcohol intake in both alcohol dependent and control animals [38]–[40]. Human imaging studies have also confirmed that alcohol induces changes in the ventral striatum. Oral administration of alcohol in humans results in robust ventral striatal blood oxygen level dependent activity, and positron emission tomography imaging shows that alcohol consumption causes endogenous dopamine and opioid release in the nucleus accumbens [41]–[43]. Genetic variation in humans has also been linked to ventral striatal activity and alcohol consumption. A recent study demonstrated an association with adolescent binge drinking and SNP rs26907 in the ras-specific guanine-nucleotide releasing factor 2 (RASGRF2) [44]. RASGRF2 is associated with ethanol-induced dopamine release in mice and the RASGRF2 haplotype containing rs26907 is associated with greater ventral striatal activity during a monetary reward anticipation task. These studies suggest that dopamine signaling may be increased in BD with acute alcohol administration.

In contrast to findings in alcohol dependence, anatomical MRI studies in cocaine and methamphetamine dependent individuals have demonstrated enlarged striatal volumes [45]–[48], although these findings are not always consistent [49]. Previous authors have suggested that the increases observed in these populations may be caused by reduced endogenous dopamine availability in the striatum [45], [46]. Supporting this hypothesis, in cases where dopaminergic signaling is decreased, such as in patients taking D2 antagonists, basal ganglia structures are enlarged, whereas in patients taking medication increasing dopamine availability, like methylphenidate for attention deficit hyperactivity disorder, striatal volumes are relatively reduced compared to unmedicated peers [50]–[52]. The greater ventral striatal volume observed here may also relate to altered dopamine signaling in BD. Studies of the unaffected family members of persons with stimulant dependence have shown that they also have enlarged striatal volumes compared to controls, suggesting its role as an endophenotypic risk factor for stimulant dependence [53]. None of the BD in this study had other substance use disorders, but our findings raise the possibility that there may be similarities between BD and subjects at risk for the development of cocaine dependence. Future studies including unaffected family members or longitudinal studies are indicated to address the role of enlarged ventral striatal volume as an endophenotypic risk factor in the development of alcohol misuse behaviours.

The findings are limited by several factors. Given the nature of our sample, it would be beneficial if these participants could have been followed longitudinally to provide information on morphological changes over time. This would also allow for comment on the possible impact of BD behaviour on continuing neuroanatomical development. Further information such as the duration of BD behaviours, length of abstinent periods between binges, and the age when BD began could provide additional insight on the unique neuroanatomical correlates of these aspects of BD. Despite these limitations, the present data provide a unique contribution to the BD literature. It is the first report of subcortical differences in college-aged BD participants not suffering from an AUD, providing new information on alcohol-related neuroanatomical changes occurring between adolescence and full adulthood. Moreover, it provides confirmation of the effects of alcohol consumption on hippocampal, and in particular left hippocampal, volume. These results highlight that even mild forms of alcohol misuse can cause changes in grey matter similar to those observed in AUD, and should reinforce the importance of early intervention.

Our findings suggest that binge drinking in young adults has different neuroanatomical correlates than those for alcohol dependence. We show that BD have greater ventral striatal volume relative to healthy volunteers. That the severity of alcohol use correlates with the opposite, a decrease in ventral striatal volume, dovetails with reports of decreased striatal volume in alcohol dependent subjects and suggests our findings are less likely due to a state effect of chronic alcohol consumption. They may instead be related to earlier binge and withdrawal exposure, compensatory mechanisms, or an endophenotypic risk factor. Further longitudinal studies including unaffected family members are warranted to address these explanatory models. Such studies might also allow identification of biological markers of subgroups at greater risk of long term negative outcomes.
